# Pharmacovigilance and Moroccan Tuberculosis Public Program: Current Situation

**DOI:** 10.1155/2014/626797

**Published:** 2014-06-12

**Authors:** Driss Soussi Tanani, Amina Tebaa, Raja Benkirane, Kenza Bennani, Ghali Iraqi, Abdelmajid Soulaymani, Rachida Soulaymani Bencheikh

**Affiliations:** ^1^Moroccan Anti Poison and Pharmacovigilance Center, Rabat 10000, Morocco; ^2^Direction of Epidemiology and Lung Diseases of Morocco, Rabat 10000, Morocco; ^3^University Hospital Moulay Youssef of Morocco, Rabat 10000, Morocco; ^4^Laboratory of Genetics and Biometry, University Ibn Tofail, Kenitra 14000, Morocco

## Abstract

The objective of this work is to demonstrate the interest of integration of pharmacovigilance in Moroccan Tuberculosis Control Program (MTCP). *Design and Data Collection.* The integration of pharmacovigilance in MTCP was conducted in October 2012 with the Global Fund support. We compared the reports notified before and after this integration (period 1: January 2010–October 2012; period 2: October 2012–December 2013). The detection of signals was based on the Information Component available in VigiMine. We used the SPSS version 10.0 and MedCalc version 7.3 for data analysis. *Results*. The average number of spontaneous reports increased from 3.6 to 37.4 cases/month (*P* < 10^−3^). The average age was 40.7 ± 17.5 years; the sex ratio was 0.8. Hepatic reactions (32.7%) predominated during the first period, while skin reactions (24.1%) were in the second period (*P* = 10^−4^), and 40.9% of cases in the first period were serious against 15.8% in second period (*P* = 0.003). Nine signals were generated (hepatic enzyme increase, cholestasis, jaundice, arthralgia, acne, lower limb edema, pruritus, skin rashes, and vomiting). *Conclusion.* The integration of pharmacovigilance in Moroccan Tuberculosis Control Program improved the management of ADRs and detected new signals of antituberculosis drugs.

## 1. Introduction

Tuberculosis (TB) remains a major public health problem globally. Clinicians treating TB patients around the world know these medicines well and are usually well aware of their associated adverse drug reactions (ADRs) [1]. The occurrence of these reactions is known to be frequent. The TB patient on treatment is taking more than one anti-TB medicine simultaneously and regimens last from many months to 2 years or more. This increases the likelihood of ADRs, some of which are severe. A recent study has shown that two-thirds of such patients have had at least one medicine stopped temporarily or permanently as a result of ADRs [2]. These events may damage public confidence in any national treatment program and affect patient adherence [3, 4]. Patients who stop taking anti-TB medicines pose a risk to themselves and to others. The generation of drug resistance is a very real risk.

Tuberculosis in Morocco remains also a public health problem with an average incidence of 83.5 cases per 10^5^ inhabitants in 2011. Resistant TB form represents 1.3% of the whole cases [5]. As public health programmes (PHPs) are extended to the more vulnerable populations such as the young, the elderly, pregnant women, and people with malnutrition, the chances of developing ADRs and interactions will increase. In addition, health practitioners and the public need more information about the potential benefit, rationality of use, and risk of the medicines given.

Recently there have been some initiatives within countries or under the leadership of WHO, to create and develop subsystems for pharmacovigilance (PV) to monitor the specific products used in their PHPs. Morocco is among the first countries which received grants from the Global Fund to strengthen pharmacovigilance in AIDS and TB [6].

The global objective of this work is to demonstrate the interest of integration of PV in Moroccan Tuberculosis Control Program (PV-MTCP). The specific objectives are as follows:

- to promote ADRs spontaneous reporting;
- to analyze and evaluate ADRs;
- to recommend regulatory action for minimization of the risks;
- to initiate studies to investigate significantly suspect reactions;
- to alert TB health practitioners, manufacturers, and the public to new signals of ADRs.

## 2. Methods

### 2.1. Study Design and Setting

It was a retrospective study (January 1, 2010–October 11, 2012) with a passive PV and without Global Fund support, compared with a prospective study (October 11, 2012–December 2013), conducted in all Moroccan TB diagnosis centers (MTDC) which are in charge of treating TB patients, after integrating PV-MTCP. These MTDC exist in all cities and receive about 27000 TB patients per year. The comparison between the 2 periods concerned the number of reports, methods used for collecting ADRs (spontaneous reporting in period 1 versus intensive pharmacovigilance reporting in period 2), nature, and seriousness of ADRs.

### 2.2. Study Population

Patients treated for TB and only who reported one or more ADRs were included in this study. TB patients who did not present ADRs were excluded from this study.

### 2.3. Data Collection

We conducted several sessions of intensive PV to report anti-TB induced ADRs for all Moroccan TB health practitioners and we validated a system of spontaneous reporting of ADRs between the MTDC and the Moroccan Pharmacovigilance Center (MPVC). Each notified case is handled by the OMS accountability method [7] that determines the relationship of cause to effect; then all cases are sent to the international database (Vigiflow) [8]. We established an electronic registry of ADRs reporting which one serves as a tool to provide answers to the different notifiers and change the information between the MPVC and them.

### 2.4. Analysis

The data were collected using Microsoft Excel software (version 5.1), and variables were described as percentage or mean (±SD). Qualitative data were compared using Chi-2 test or Fisher exact test. Quantitative data were compared using Student's *t*-test. The statistical significance level was set at *P* < 0.05. Analysis was performed using SPSS (version 10.0) and MedCalc (version 7.3) Software.

The detection of signals was based on the Information Component (IC) available in Uppsala Monitoring Center (UMC) software VigiMine [9], method of Bayesian Confidence Propagation Neural Network used by UMC for automatic generation of signals:

(1) $$\begin{array}{c} \text{IC}={\text{Log}}_{2}\left(\frac{\text{Observed}\;\text{ADRs}\;\text{probability}}{\text{Expected}\;\text{ADRs}\;\text{probability}}\right). \end{array}$$

Adverse drug reaction is considered a signal if the IC_025_ > 0: the probability of observed ADRs is greater than the probability of expected ADRs (IC_025_: Information Component within a range of 95% confidence).

## 3. Results

The international database VigiSearch [9] during the global period of study (January 2010–December 2013) showed that Morocco with an average TB incidence (50–100 cases/10^5^ inhabitants) has recorded 927 ADRs (Table 1).

### 3.1. Descriptive Study: General Information

608 cases were reported (927 ADRs) during the study (January 2010–December 2013). The average age of patients was 40.7 ± 17.5 years with a sex ratio of 0.8. The most prescribed anti-TB drug was ERIP-K4 (83.7%) since it contains 4 combined anti-TB drugs (Ethambutol, Rifampicin, Isoniazid, and Pyrazinamide) followed by Riniazide 10.1% (Rifampicin, Isoniazid) as maintenance treatment. Accountability of cases according to the WHO method showed that 5% had a certain relationship of cause to effect, 15% had a probable relationship, and 80% had a possible relationship. 140 of the cases were serious (23%) with 8 deaths (1.3%), 21 cases had a commitment prognosis (3.4%), 4 cases developed sequelae (0.6%), and 107 cases required hospitalization or prolongation of hospitalization (17.5%). The outcome was favorable in 47.2% of cases, 16.2% were healing, 35.2 were unknown, and 1.3% died.

For fatal cases, there were 3 women aged between 20 and 29 years: TB MDR with lower limb edema, lymph node TB in a pregnant woman with fulminant hepatitis, and pulmonary TB with toxic epidermal necrolysis; there were 5 men aged between 28 and 70 years: TB MDR with lower limb edema, pulmonary TB with hepatic encephalopathy, multifocal TB with cytolytic hepatitis, pulmonary TB with cholestatic hepatitis, and TB + heart failure with fulminant hepatitis (Table 2).

### 3.2. Descriptive Study: Nature of ADRs (Table 3)

**Figure 1:**
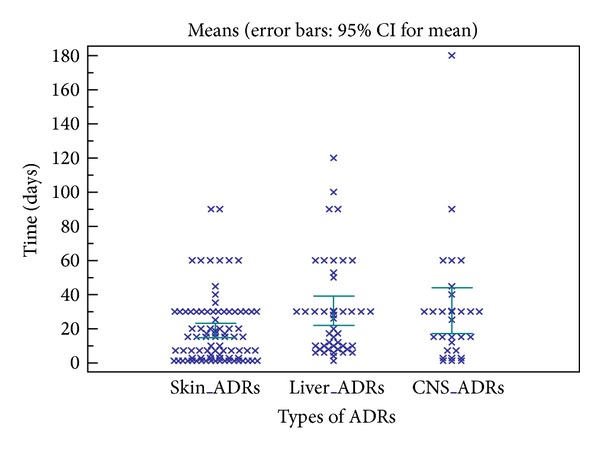
Comparison of average times to onset of cutaneous, hepatic, and neurological ADRs.

The majority of ADRs occurred during the first month after starting treatment with a difference depending on the nature of the ADRs (Figure 1). ADRs of liver and biliary system disorders (32.8%) predominated in period 1 with cytolytic hepatitis as the most predominant symptom followed by skin and appendages disorders (26.3%) with pruritus as the most predominant symptom, while ADRs of skin and appendages' disorders predominated in period 2 (24.2%) followed by ADRs gastrointestinal system disorders (21%) with epigastric pain and vomiting as the most predominant symptom (Table 3).

### 3.3. Analytic Study

We compared the notification before and after PV-MTCP. The average number of reports increased from 3.6 to 37.4 cases/month (*P* < 10^−3^). The System Organ Class of ADRs reported during the first period concerned mainly liver and biliary system disorders (32.8%) because prescribers were reporting mainly serious ADRs, while skin and appendage system disorders (24.2%) of ADRs were predominantly reported in the second period.

New ADRs occurred in period 2 as ADRs of hearing and vestibular system disorders, respiratory system disorders, endocrine system disorders, visual system disorders, and reproductive system disorders (Table 3).

The comparison of seriousness cases before and after integration of PV-MTCP showed that in period 1 there were more hospitalization (28.9%) and development life-threatening (9.6%) (Table 4). The comparison of ADRs outcome before and after integration of PV-MTCP showed that in period 2 the outcome of ADRs was more favorable with less unknown cases but without significant difference (Table 5).

### 3.4. Signals Detection

The signal detection was focused on the cases related to combined anti-TB form (ERIP-K4) recorded in VigiMine. We found 875 ADRs related to this combination; 268 of them were issued from Morocco (30.6%). 18 international signals have been generated; 11 of them were from Morocco (Table 6).

## 4. Discussion

The international database (Vigiflow) during the period of study (January 2010–December 2013) showed that Morocco with an average TB incidence (50–100 cases/10^5^ inhabitants) has recorded 608 cases (927 ADRs). 176 cases were reported (327 ADRs) before PV-MTCP (January 2010–October 2012) and 432 cases were reported (600 ADRs) after PV-MTCP (October 2012–December 2013). USA is the best notifier country (1076 ADRs) with a low incidence (<24/10^5^ inhabitants) and South Africa is the worst notifier country (191 ADRs) with the high incidence (>300/10^5^ inhabitants). However, Republic of Korea and India have the highest rate of notifications (4650 and 2960 ADRs, resp.) due to their high incidence (100–300 cases/10^5^ inhabitants), Table 1.

This increase of ADRs in period 2 was due to the effective integration of PV in the MTCP with awareness of the majority of anti-TB prescribers and involvement for spontaneous reporting of TB ADRs and feedback with practical procedures of ADRs management are regularly sent to anti-TB prescribers to motivate them to reporting ADRs.

In our study, females had a higher incidence of ADRs. In general, females are at a higher risk of developing ADRs [10]. It might be because they pass through life stages like pregnancy, menarche, and so forth, which modify the drug response [11]. Studies from UK and Canada also reported females to have a significantly higher incidence of ADRs due to anti-TB drugs [12, 13]. This suggests the need for special precautions while prescribing anti-TB drugs to females.

We compared the average time of onset of cutaneous, hepatic, and neurological ADRs. Cutaneous ADRs appeared first with an average time of 18.9 ± 20.0 days. Hepatic and neurological ADRs are with almost identical average time (30.5 ± 28.4 and 30.4 ± 36.0 days) but with a wide standard deviation for neurological ADRs (Figure 1). Cutaneous ADRs appeared first because their mechanism is often immunoallergic against hepatic ADRs and neurological ADRs take more time to appear. The majority of ADRs occurred during the first month after starting treatment requiring more vigilance during this period regardless of the nature of ADRs.

Onset of the ADRs is an important factor helpful in early detection of the ADRs. Also in studies from India [14] and from Nepal [15] more than half of ADRs occurred within the first 30 days after starting TB treatment. It is essential for the healthcare professionals to counsel the patients regarding the early identification of ADRs in the first few weeks. Regular monitoring of the patients during these initial weeks might be essential for early detection of ADRs.

On the severity of ADRs, cases of period 2 were significantly less severe than period 1 (15.8% versus 40.9%, *P* < 0.001) with less hospitalization (13% versus 28.9%, *P* < 0.001) and less development life-threatening (1% versus 9.6%, *P* < 0.001). Eight patients died (1.3%) with an average age of 36.8 ± 18.0 years and a male sex ratio = 1.6, among which 5 died by hepatic complications, 2 had a multiresistant TB, and one died by Lyell syndrome (Table 2). Twenty-one cases (3.4%) had ADRs development life-threatening, and 4 cases developed sequelae 0.6% (2 left deafness, 2 ataxo-spasmodic diseases), Table 4.

In Morocco, the average of TB incidence has stagnated during recent years, about 81 cases per 100000 inhabitants [16]; then the increase of ADRs during period 2 is mainly due to the integration PV-MTCP. The reporters during the first period were in the majority from university and provincial hospitals, but in the second period they were mainly from MTDC. Among the 16 regions of Morocco, 7 were involved in the reporting of ADRs in the first period, whereas 14 regions were engaged in the second period. The System Organ Class of ADRs reported during the first period concerned mainly liver and biliary system disorders because the physicians reported mainly serious ADRs as hepatitis, while skin and appendage system disorders of ADRs were predominantly reported in the second period. This second period is quite rich in ADRs because it has been active pharmacovigilance which forced prescribers to notify all ADRs. There was a significant increase of reports in gastrointestinal, general, musculoskeletal, and psychiatric system disorders between 2 periods but a significant decrease of reports in metabolic system disorders because the notifications during the first period emanated mainly from university hospitals (Table 3).

There were new ADRs of reproductive, vision, respiratory, hearing, and vestibular and endocrine disordersnotified in the second periodafter PV-MTCP reflecting the interest of active pharmacovigilance.

The comparison of ADRs evolution before and after integration of PV-MTCP showed that in period 2 the outcome of ADRs was more favorable (52.7% versus 41.8%) with less unknown cases (29% versus 41.4%) but without significant difference (Table 5).

The most common system affected by the ADRs in our study (PV-MTCP) was skin and appendage (24.2%). Also in 2 studies from Thailand and Malaysia, skin and appendage system was the most affected (48.9 and 49.5%) [17, 18]. In an Indian study, the majority of the patients (53%) had gastrointestinal reactions [14]. In 2 studies from Nepal and Iran [15, 19], the most common system affected by the ADRs was liver and biliary system (58.5 and 37%).

The principal clinical risk factors for hepatotoxicity are old age, malnutrition, alcoholism, HIV infection, and chronic hepatitis B and C infections [20]. There are several strategies to prevent the occurrence of these ADRs. Drug-induced hepatic dysfunction usually occurs within the initial few weeks of the intensive phase of anti-TB chemotherapy [20]. It is also recommended that liver function should be studied every two weeks during ATT to prevent serious hepatotoxicity [21]. A few guidelines were also published mentioning the management of hepatotoxicity due to anti-TB drugs [22, 23]. It is also the responsibility of the health care professionals to counsel the patients regarding the early signs of hepatotoxicity.

For minimizing risk of serious ADRs, the MPVC collaborating with some hepatologists and phthisiologists developed a practical procedure of TB hepatotoxicity that helps prescribers to manage the risks associated with anti-TB drugs.

Adverse drug reactions to certain drugs may differ within each country, reflecting different patterns of prescription, socioeconomic status, and culture.

On December 31, 2013, 18 international signals have been generated with combined anti-TB form (ERIP-K4). Among these 18 signals, 11 were from Morocco including 3 critical signals: hepatitis, cholestatic hepatitis, and peripheral neuropathy. Three signals had a Moroccan score of IC_025_ higher than international score of IC_025_: arthralgia, pruritus, and peripheral oedema, testifying to the importance of these three signals (Table 6).

All these Moroccan signals are known except peripheral edema including lower limb edema which is a new signal not documented in the literature.

Therefore, a technical committee of pharmacovigilance met in July 2013 to discuss these signals. Recommendations were issued for increased vigilance of these signals especially lower limb edema requiring more laboratory investigations to rule out other causes of the occurrence of edema. The committee decided also to initiate a study to evaluate the relationship of accountability of this significant lower limb edema.

*Limitations*. The strong point of our study is the collection of all major and minor ADRs, but the limitation is the absence of files of patients who have not developed ADRs for estimating the incidence of ADRs.

## 5. Conclusion

The integration of pharmacovigilance in the Moroccan Tuberculosis Control Program has increased spontaneous reporting of all TB ADRs, decreased severity of ADRs, allowed a procedure of anti-TB drugs induced-hepatotoxicity and detected of new signals of anti-TB drugs.

## Figures and Tables

**Table 1:** International database VigiSearch (January 2010–December 2013).

Country	Number of reports	TB incidence (/10^5^)
Republic of Korea	4650	100–300
India	2960	100–300
USA	1076	<24
Morocco	927	50–100
France	302	<24
South Africa	191	>300
Russian Federation	70	100–300
Tunisia	85	<24

**Table 2:** Characteristics of eight dead cases.

Age (year)	Sex	Indication	ADR	Accountability
20	F	Multiresistant TB	Lower limb edema	Possible
30	M	Multiresistant TB	Lower limb edema	Possible
28	M	Pulmonary TB	Hepatic encephalopathy	Probable
70	M	Multifocal TB	Cytolytic hepatitis	Possible
44	M	Pulmonary TB	Cholestatic hepatitis	Possible
—	M	TB + heart failure	Hepatitis	Possible
29	F	Lymph node TB + pregnancy	Fulminant hepatitis	Probable
27	F	Pulmonary TB	Lyell syndrome	Possible

**Table 3:** Comparison of ADRs types before and after integration of PV-MTCP.

System Organ Class (disorders)	Period 1 *n* (%)	Period 2 *n* (%)	*P*
Skin and appendages disorders	86 (26.3)	145 (24.2)	NS
Gastrointestinal system disorders	29 (8.9)	126 (21)	0,007
Liver and biliary system disorders	107 (32.8)	87 (14.5)	NS
General disorders	12 (3.7)	86 (14.4)	0,0007
Central and peripheral nervous system	42 (12.9)	46 (7.7)	NS
Musculoskeletal system disorders	8 (2.4)	36 (6)	0,03
Psychiatric disorders	6 (1.8)	20 (3.4)	0,04
Hearing and vestibular disorders	0	10 (1.7)	—
Respiratory system disorders	0	10 (1.7)	—
Metabolic disorders	21 (6.4)	9 (1.5)	0,02
Platelet, bleeding, and clotting disorders	5 (1.5)	6 (1)	NS
Endocrine disorders	0	6 (1)	—
Vision disorders	0	5 (0.8)	—
Heart rate and rhythm disorders	2 (0.6)	3 (0.5)	NS
White cell disorders	2 (0.6)	2 (0.3)	NS
Red blood cell disorders	2 (0.6)	1 (0.1)	NS
Urinary system disorders	5 (1.5)	1 (0.1)	NS
Reproductive disorders	0	1 (0.1)	—

Total	327 (100)	600 (100)	

NS: not significant.

**Table 4:** Comparison of seriousness cases before and after integration of PV-MTCP.

Seriousness	Period 1 *n* (%)	Period 2 *n* (%)	*P*
Hospitalization/prolonged	51 (28.9)	56 (13)	<0,001
Life-threatening	17 (9.6)	4 (1)	<0,001
Sequelae	2 (1.2)	2 (0.4)	NS
Death	2 (1.2)	6 (1.4)	NS

Total	72 (40.9)	68 (15.8)	<0,001

NS: not significant.

**Table 5:** Comparison of cases evolution before and after integration of PV-MTCP.

Evolution	Period 1 (%)	Period 2 (%)	*P*
Favorable	41.8	52.7	NS
Ongoing	15.6	16.9	NS
Unknown	41.4	29	NS
Lethality	1.2	1.4	NS

Total	100	100	

NS: not significant.

**Table 6:** Moroccan signals with anti-TB combined form.

Nature of Signal	International IC_025_	Moroccan IC_025_
Hepatitis*	3.69	0.12
Increase hepatic enzymes	2.85	2.79
Jaundice	2.68	1.25
Cholestatic hepatitis*	2.16	1
Acne	1.54	0.67
Arthralgia	1.54	2.54
Vomiting	1.33	0.76
Pruritus	1.14	1.78
Abdominal pain	0.60	0.33
Periperal neuropathy*	0.41	0.18
Peripheral edema	0.14	1.98

*Critical ADRs signal.
